# Mitogen-activated protein kinases (MAPKs) regulate IL-6 over-production during concomitant influenza virus and *Staphylococcus aureus* infection

**DOI:** 10.1038/srep42473

**Published:** 2017-02-14

**Authors:** Carolin Klemm, Christin Bruchhagen, Andre van Krüchten, Silke Niemann, Bettina Löffler, Georg Peters, Stephan Ludwig, Christina Ehrhardt

**Affiliations:** 1 Institute of Virology Muenster (IVM), Westfaelische Wilhelms-University Muenster, Von Esmarch-Str. 56, D-48149 Muenster, Germany; 2 Institute of Medical Microbiology, University Hospital of Muenster, Domagkstr, 10, D-48149 Muenster, Germany; 3 Institute of Medical Microbiology, University Hospital Jena, Erlanger Allee 101, D-07747 Jena, Germany; 4 Cluster of Excellence Cells in Motion (CIM), University of Muenster, Muenster, Germany

## Abstract

Bacterial super-infections are a major complication of influenza virus (IV) infections and often lead to severe pneumonia. One hallmark of IV-associated *Staphylococcus aureus (S. aureus*) infection is rapid progression to a serious disease outcome. Changes in immune and inflammatory host responses increase morbidity and complicate efficient therapy. A key player during inflammation is the multifunctional cytokine IL-6. Although increased IL-6 levels have been observed after severe disease upon IV and/or bacterial super-infection, the underlying molecular mechanisms still remain to be elucidated. In the present study, we focused on cellular signalling pathways regulating IL-6 production upon IV/*S. aureus* super-infection. Additionally, infection with viable bacteria was mimicked by lipoteichoic acid stimulation in this model. Analyses of cellular signalling mechanisms revealed synergistically increased activation of the MAPK p38 as well as enhanced phosphorylation of the MAPKs ERK1/2 and JNK in the presence of super-infecting bacteria. Interestingly, inhibition of MAPK activity indicated a strong dependence of IL-6 expression on p38 and ERK1/2, while the MAPK JNK seems not to be involved. Thus, our results provide new molecular insights into the regulation of IL-6, a marker of severe disease, which might contribute to the lethal synergism of IV and *S. aureus*.

Bacterial super-infections, notably those caused by *Staphylococcus aureus (S. aureus*), contribute to the morbidity and mortality of influenza virus (IV) infections^1^,^2^,^3^,^4^,^5^. In recent years methicillin-resistant *S. aureus* (MRSA) strains have spread in communities and caused infections even in healthy and fully immunocompetent individuals^6^. Accordingly, IV-associated cases of staphylococcal pneumonia that are characterised by rapid progression to severe disease commonly appear in the population^7^. A prominent characteristic of IV/*S. aureus* super-infection compared with other bacterial invaders is the timing of infection. *S. aureus* is mostly detected as a secondary invader during concomitant viral-bacterial infection^8^,^9^, whereas an infection with *Streptococcus pneumoniae (S. pneumoniae*) occurs at late phases following the viral clearance^3^,^8^,^9^,^10^.

Several mechanisms have been described that contribute to the fatal synergism of IV infection and bacterial super-infection. During the progression of an IV infection epithelial damage changes the functionality of the lung leading to increased receptor availability, which promotes bacterial attachment. Additionally, the functionality of different immune cell populations, such as macrophages or neutrophils, is modulated by IV infection and hampers efficient bacterial removal by the innate immune system. In consequence, clearance of bacteria is delayed and virus titres increase. Obvious changes in the immune response of the host contribute to disease severity and as a result lung damage can occur without effective pathogen control^9^.

The inflammatory response is induced after recognition of pathogen-associated molecular patterns (PAMPs) of IV and/or *S. aureus*, such as viral RNA, bacterial DNA or bacterial cell wall components like lipoteichoic acid (LTA). These molecules are ligands for pattern recognition receptors (PRRs) like the cytosolic RIG-I family of RNA helicases or the membrane-bound toll-like receptor (TLR) family, to name but a few. Binding to specific receptors triggers an innate immune response and leads to the synthesis of inflammatory mediators. Findings from *in vivo* super-infection models show a stronger boost of inflammatory cytokines and chemokines compared with animals infected with only one pathogen, resulting in increased morbidity and mortality^11^,^12^. Even with appropriate and timely antibiotic administration, the inflammatory response is enhanced after super-infection and fatal outcomes may occur^3^,^10^,^13^. These observations suggest that beside higher levels of pathogen load, super-infection leads to a dysregulated immune response that contributes to the severe outcome of disease. However, little is known about the underlying molecular signalling mechanisms regulating the expression of inflammatory cytokines and chemokines in a super-infection scenario.

Upon pathogen recognition, the onset of inflammation acts as an early nonspecific protective mechanism to limit pathogen spread, and to attract and activate immune cells. As a first line of defence the type I interferon (IFN) response is most effective against IV infections^14^. However, *in vitro* data suggest that this system is blocked by *S. aureus* super-infection, leading to enhanced viral replication^15^. Another important regulator of immune response and inflammation is the multifunctional cytokine IL-6. Elevated levels of this inflammatory cytokine are associated with severe clinical disease after IV infection^16^,^17^,^18^,^19^,^20^,^21^,^22^,^23^. Furthermore, IL-6 is massively expressed after super-infection in *in vitro* and *in vivo* models. These effects are higher than the sum of both single pathogen infections^11^,^12^,^24^. IL-6 is synthesised in various cell types, including different kinds of immune cells as well as in fibroblasts and endothelial cells. Due to the unique receptor system for IL-6, it acts on almost all cells and has multiple biological functions^25^,^26^. One main function is the induction of the hepatic acute-phase response (APR), characterised by production and secretion of acute-phase proteins (APPs), such as Serum Amyloid A (SAA), Haptoglobin (Hp), Orosomucoid (ORM) and γ-Fibrinogen (FGG)^27^,^28^,^29^.

Induction and regulation of the inflammatory response is controlled, among other mechanisms, by mitogen-activated protein kinases (MAPKs), a well-studied, important family of kinases. MAPK signalling pathways encompass cascades of kinases, which convert extracellular signals into cellular responses^30^. In mammalian cells, isoforms of the MAPKs p38, ERK1/2 and JNK are the best-studied members of the family^31^. Upon pathogen challenge, MAPKs are activated, leading to the expression of inflammatory cytokines and chemokines^32^,^33^,^34^. A dysregulated inflammatory response after viral or bacterial infection occurs in cases of septicaemia or infection with highly pathogenic avian IV subtypes (HPAIV), resulting in massive over-production of pro-inflammatory cytokines and chemokines. This so called “cytokine storm” has been linked to the activity of the MAPK family members p38 and ERK1/2^35^,^36^. Additionally, MAPKs fulfil pathogen-supportive roles, during both viral and bacterial infection. Activation of the Raf/MEK/ERK signalling pathway facilitates IV replication by inducing nuclear export of viral ribonucleoprotein complexes (RNPs) at late stages of the viral life cycle^37^. Furthermore, *S. aureus* modulates the inflammatory response of the host and induces anti-inflammatory cytokines by selective signalling through the MAPK ERK1/2^38^.

The activity of MAPKs is controlled by kinases and phosphatases. Protein phosphatase 2A (PP2A) is a ubiquitously expressed serine/threonine phosphatase that exists as a tri-molecular complex consisting of a structural subunit (A subunit), a variable regulatory subunit (B subunit) and a catalytic subunit (C subunit)^39^. PP2A controls multiple inflammatory signalling pathways by dephosphorylating kinases such as MAPKs p38, ERK1/2 and JNK^40^.

In this study, we investigated the regulation of the multifunctional cytokine IL-6 after IV/*S. aureus* super-infection in human lung epithelial cell lines. Using an *in vitro* super-infection model^15^, we observed a synergistically enhanced expression of IL-6 as well as other pro-inflammatory cytokines and chemokines. Concurrently, the activation of the MAPKs p38, ERK1/2 and JNK was increased. Our data indicate a strong correlation between enhanced activity of the MAPKs p38 and ERK1/2 and an over-production of *IL*-*6* in the presence of both pathogens. Furthermore, cytokine over-production upon IV/*S. aureus* super-infection increases the pro-inflammatory response at the level of APPs *in vitro* and *in vivo*. Here, we show a molecular mechanism responsible for a dysregulated inflammatory response during a concomitant IV/*S. aureus* infection scenario.

## Results

### IV-induced levels of inflammatory cytokines and chemokines are increased upon super-infection with *S. aureus*

**Figure 1:**
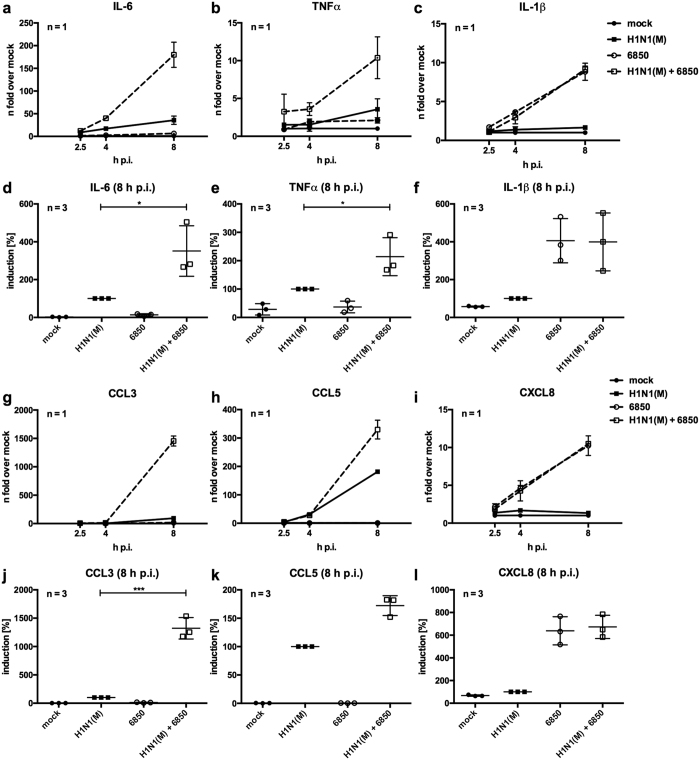
IV-induced mRNA levels of pro-inflammatory cytokines and chemokines are increased upon super-infection with *S. aureus*. Calu-3 cells were infected with IV H1N1(M) (MOI 5) for 0.5 h and super-infected with *S. aureus* 6850 (MOI 50). In 8 h incubations, extracellular bacteria were removed by gentamicin treatment 3 h post bacterial infection. Levels of *IL*-*6, TNFα, IL*-*1β, CCL3, CCL5* and *CXCL8* mRNA were measured in duplicates at 2.5 h, 4 h and 8 h p.i. One representative line graph (mean ± SD) over time is depicted to show n-fold induction over mock-infected cells (**a**–**c**,**g**–**i**). For 8 h p.i. means ± SD of three independent experiments are shown. Each symbol represents an individual value. Levels of IV-infected samples were arbitrarily set as 100% (**d**–**f**,**j**–**l**). After normalisation, two-tailed unpaired t-tests were performed for comparison of IV H1N1(M)-infected and IV H1N1(M)/*S. aureus* 6850 super-infected samples at 8 h p.i. (*p < 0.05, **p < 0.01, ***p < 0.001).

Increased inflammation contributes to the severe outcome of *S. aureus* super-infections following IV^8^,^41^. Up-regulation of certain inflammatory mediators has been linked to serious disease progression, in case of IV infection this has been shown for the multifunctional cytokine IL-6^16^,^17^,^18^,^19^,^20^,^21^,^22^,^23^. To investigate the molecular changes of combined IV and *S. aureus* infection on cytokine expression, we employed an *in vitro* super-infection model^15^. The lung is the primary site of IV replication and responds to infection by secreting inflammatory cytokines and chemokines. Thus, we infected human lung epithelial cells (Calu-3) with IV H1N1(M) for 30 min to allow virus attachment and internalisation. Cells were washed and incubated with *S. aureus* 6850 for 3 h to super-infect the majority of cells. Then, non-internalised bacteria were removed by an antibiotic wash step. Analysis of mRNA levels of *IL*-*6* and other pro-inflammatory cytokines and chemokines at 2.5 h, 4 h and 8 h post infection (p.i.) (Fig. 1) revealed a moderate induction of *IL*-*6, TNFα, CCL3* and *CCL5* upon IV infection of Calu-3 cells (Fig. 1a,b,g,h). In contrast, *S. aureus* 6850 infection alone resulted in a marginal induction of these cytokines and chemokines, while *IL*-*1β* and *CXCL8* mRNA levels were elevated after bacterial infection (Fig. 1c,i). After super-infection, mRNA levels of *IL*-*6, TNFα, CCL3* and *CCL5* were substantially increased, especially at 8 h p.i. (Fig. 1d,e,j,k). The mRNA levels of *IL*-*1β* and *CXCL8* that were induced by bacterial infection were not changed in the presence of both pathogens (Fig. 1c,f,i,l). As we detected a synergistic effect on *IL*-*6* expression upon IV/*S. aureus* super-infection in our *in vitro* infection model (Fig. 1a,d) and high expression of IL-6 has been previously linked to severe disease^16^,^17^,^18^,^19^,^20^,^21^,^22^,^23^, we focused on the regulation of this cytokine in further experiments.

To confirm the release of IL-6 into the culture supernatant of infected Calu-3 cells, conditioned medium was transferred onto hepatocytes. Treatment of HepG2 cells with this conditioned medium from infected Calu-3 cells provoked in case of IV/*S. aureus* super-infection a significant difference in mRNA levels of the IL-6 dependent APPs *SAA* and *ORM* (Supplementary Fig. S1a,b). Furthermore, a similar tendency was seen for *FGG* mRNA levels, but not for *Hp* mRNA levels (Supplementary Fig. S1c,d).

**Figure 2:**
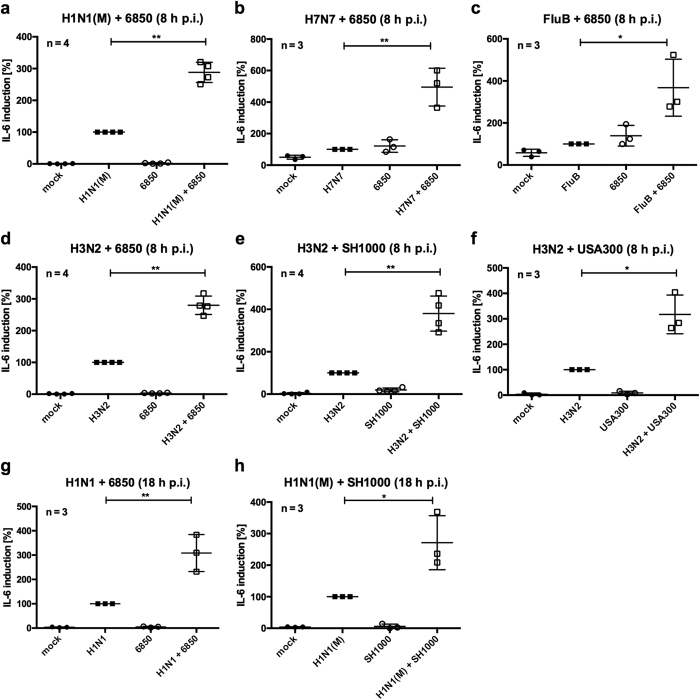
*IL*-*6* hyper-induction during super-infection is IV and *S. aureus* strain independent. A549 cells were infected with IV H1N1(M) (MOI 5 (**a**), MOI 0.5 (**h**)), H7N7 (**b**), FluB (**c**), H3N2 (**d**–**f**) (MOI 5) or H1N1 (MOI 0.5 (**g**)) for 0.5 h and super-infected with *S. aureus* 6850 (MOI 50 (**a**–**d**), MOI 0.1 (**g**)), SH1000 (MOI 50 (**e**), MOI 0.1 (**h**)) or MRSA USA300 (MOI 50 (**f**)). Extracellular bacteria were removed by antibiotic treatment 3 h post bacterial infection. *IL*-*6* mRNA levels were measured at 8 h p.i. (**a**–**f**) or 18 h p.i. (**g**,**h**). Means ± SD of at least three independent experiments are shown. Levels of IV-infected samples were arbitrarily set as 100%. After normalisation, two-tailed unpaired t-tests were performed for comparison between IV-infected and IV/*S. aureus* super-infected samples (*p < 0.05, **p < 0.01).

Further experiments using the human epithelial cell line A549 verified synergistically elevated *IL*-*6* mRNA levels at different time points upon super-infection with IV H1N1(M), H7N7, FluB, H3N2 or H1N1 and the *S. aureus* strains 6850, SH1000 or MRSA USA300 compared to single pathogen-infected cells (Fig. 2). This effect on *IL*-*6* expression was not limited to human epithelial cells. In primary human endothelial cells (HUVEC), which were described as strong producers of IL-6 upon infection^42^, we detected a synergistical effect on *IL*-*6* levels during IV/*S. aureus* super-infection (Supplementary Fig. S2a–c). Comparable results were also detected in human monocytic THP-1 cells as well as in the murine cell lines LLC and MEF (Supplementary Fig. S2d–h).

To exclude that the observed increase in *IL*-*6* mRNA levels is due to a higher pathogen load upon IV/*S. aureus* super-infection, mRNA levels of the viral matrix protein 1 (*M1*) and the bacterial Shikimate dehydrogenase (*aroE*) were investigated by RT-qPCR. No significant differences between single and super-infected samples were detected in Calu-3, A549, THP-1 or HUVEC cells (Supplementary Fig. S3).

**Figure 3:**
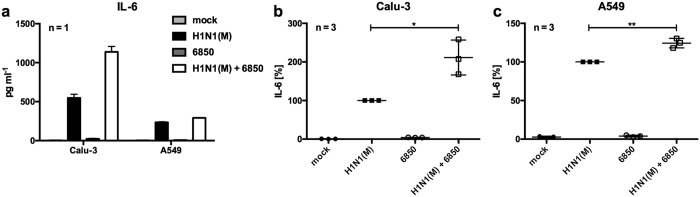
IV/*S. aureus* super-infection increases IL-6 secretion in Calu-3 and A549 cells. Calu-3 (**a**,**b**) or A549 (**a**,**c**) cells were infected with IV H1N1(M) (MOI 5) for 0.5 h and super-infected with *S. aureus* 6850 (MOI 50). Extracellular bacteria were removed by gentamicin treatment 3 h after bacterial infection. At 8 h p.i. cell supernatants were collected and secreted IL-6 was determined in duplicates by ELISA. One representative experiment (mean + SD) is depicted to show the quantitative amounts of IL-6 (**a**). Means ± SD of three independent experiments are shown, where IV-infected samples were arbitrarily set as 100% (**b**,**c**). After normalisation, two-tailed unpaired t-tests were performed for comparison between IV H1N1(M)-infected and IV H1N1(M)/*S. aureus* 6850 super-infected samples (*p < 0.05, **p < 0.01).

To verify IL-6 over-production after super-infection at the protein level, an ELISA was performed to detect secreted IL-6 in culture supernatants of Calu-3 and A549 cells (Fig. 3). Infection with *S. aureus* 6850 resulted in marginal production of IL-6. While infected Calu-3 cells secreted more IL-6 into the supernatant than A549 cells 8 h p.i. (Fig. 3a), both cells lines showed a synergistic effect on IL-6 secretion upon super-infection (Fig. 3b,c).

Thus, IV/*S. aureus* super-infection increases levels of the multifunctional cytokine IL-6 in human and murine cells independent of the IV and *S. aureus* strain.

### Co-stimulation with the *S. aureus* cell wall component LTA increases IV-induced *IL*-*6* mRNA levels

In further analyses, we investigated the molecular regulation of IL-6 expression, which might indicate important signalling events responsible for the hyper-induction of inflammation during IV/*S. aureus* super-infection.

**Figure 4:**
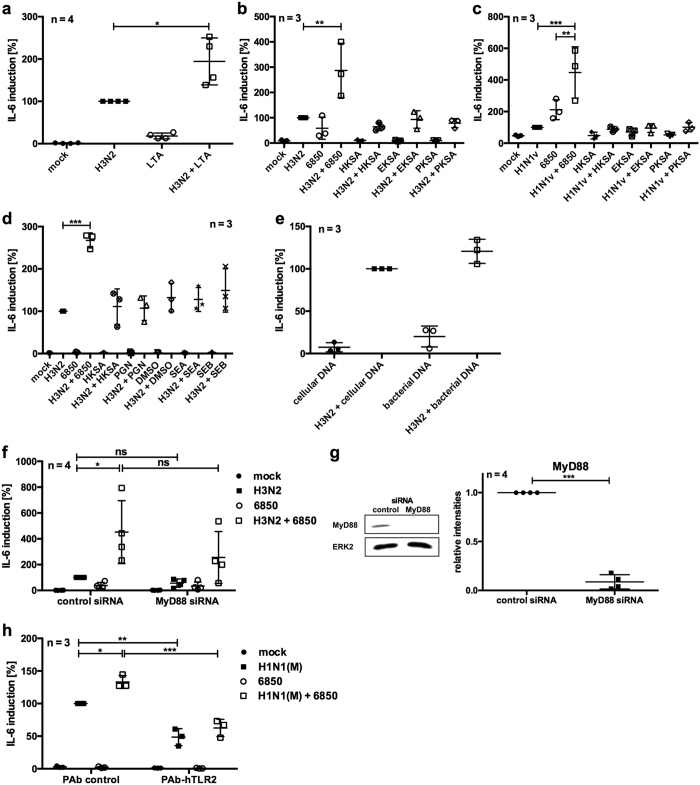
Co-stimulation with TLR2 ligand LTA increases IV-induced *IL*-*6* mRNA levels. A549 cells were infected with IV H3N2 (**a**,**b**,**d**–**f**) or H1N1v (**c**) (MOI 5) for 0.5 h and super-infected with *S. aureus* 6850, HKSA, EKSA or PKSA (MOI 50) (**b**–**d**,**f**) or co-stimulated with 5 μg ml^−1^ LTA (**a**), 5 μg ml^−1^ PGN, 0.5 μg ml^−1^ SEA, 0.5 μg ml^−1^ SEB, solvent DMSO (**d**), 10 μg ml^−1^ cellular or bacterial DNA (**e**) for 7.5 h. Extracellular bacteria were removed by gentamicin treatment 3 h post bacterial infection (**b**–**d**,**f**). Prior to infection, cells were transfected with 30 nM siRNA directed against MyD88 or control siRNA and incubated for 48 h (**f**,**g**). Protein expression was monitored by Western blot analysis (original blots are depicted in Supplementary Fig. S8a) and quantified by using ImageJ 2006.02.01 software. Means ± SD of four independent experiments are shown (**g**). A549 cells were infected with IV H1N1(M) (MOI 5) for 0.5 h and incubated for 3 h in DMEM/INV. Five μg ml^−1^ neutralising TLR2 antibody or isogenic control were added and cells were incubated for 0.5 h. Subsequently, *S. aureus* 6850 (MOI 50) was directly added to the cells for 4 h (**h**). *IL*-*6* mRNA levels were measured at 8 h p.i. (**a**–**f**,**h**). Means ± SD of at least three independent experiments are shown. IV-infected (control) samples were arbitrarily set as 100%. After normalisation, two-tailed unpaired t-tests were performed for comparison between IV H3N2-infected and LTA co-stimulated samples (**a**) or control and treated cells (**g**) and one-way ANOVA followed by Dunnett’s multiple comparison tests were used to compare IV-infected and super-infected samples (**b**–**d**) or control and treated cells (**f**,**h**) (*p < 0.05, **p < 0.01, ***p < 0.001).

We aimed to elucidate whether viable *S. aureus* is required for the over-production of IL-6 or if individual bacterial PAMPs may be sufficient to provoke this response. For this reason, the bacterial cell wall components LTA and PGN as well as bacterial DNA were tested in a co-stimulation scenario in A549 cells (Fig. 4a,d,e). Additionally, heat- (HKSA), paraformaldehyde- (PKSA) or ethanol-killed *S. aureus* (EKSA) as well as staphylococcus enterotoxins SEA and SEB were tested for their ability to increase IV-induced *IL*-*6* levels (Fig. 4b–d).

None of the indicated components was able to induce strong expression of *IL*-*6* by themselves (Fig. 4). However, LTA stimulation of virus-infected cells resulted in similar *IL*-*6* mRNA levels as induced by genuine super-infection with living *S. aureus* (Fig. 4a). But, co-stimulation with the other tested components hardly increased IV-induced *IL*-*6* levels (Fig. 4b–e).

To unravel the importance of the TLR2 ligand LTA in a super-infection scenario, expression of the intracellular TLR adaptor protein myeloid differentiation primary response gene 88 (MyD88) was knocked-down using siRNA interference (Fig. 4f). Detectable MyD88 protein levels were more than 90% reduced upon siRNA treatment (Fig. 4g). Decreased MyD88 expression resulted in an obvious reduction of *IL*-*6* mRNA levels upon IV/*S. aureus* super-infection compared with control cells (Fig. 4f). Furthermore, blocking of TLR2-mediated signalling by the use of neutralising antibodies significantly reduced *IL*-*6* expression after infection of A549 cells (Fig. 4h).

Based on these results, it can be concluded that LTA-mediated signalling through TLR2 is a major pathway that provokes a strong *IL*-*6* induction upon *S. aureus* super-infection of IV-infected A549 cells.

### IV-induced phosphorylation of MAPKs p38, ERK1/2 and JNK is increased upon super-infection with *S. aureus*

Levels of inflammatory cytokines have to be tightly regulated to sustain a particular physiological state. To unravel the molecular mechanisms that control hyper-induction of IL-6 during IV/*S. aureus* super-infection, we explored the potential impact of *IL*-*6* mRNA stability and the role of IFNβ as a major inducer of innate immune responses as well as the involvement of different signalling pathways.

To study the potential impact of super-infection on *IL*-*6* mRNA stability, the decay of *IL*-*6* mRNA in the presence of the transcription inhibitor actinomycin D (ActD) was investigated. A549 cells were super-infected with IV H1N1(M) and *S. aureus* 6850. At 6 h p.i. ActD was added to the cells and, subsequently, *IL*-*6* mRNA was measured by RT-qPCR. However, neither infection with a single pathogen nor super-infection with IV/*S. aureus* resulted in altered mRNA stability of *IL*-*6* (Supplementary Fig. S4).

Increased IL-6 levels might be a side effect of enhanced IFNβ synthesis during IV/*S. aureus* super-infection^15^. Previous *in vitro* data show that dendritic cells secrete more IL-6 upon challenge with *S. pneumoniae* after priming with type I IFN^24^. In this context, A549 cells were infected with *S. aureus* 6850 and co-stimulated with recombinant human IFNβ. Despite increased *IL*-*6* mRNA levels after IFNβ stimulation, super-infection with *S. aureus* 6850 did not additionally increase IFNβ-induced *IL*-*6* synthesis (Supplementary Fig. S5).

**Figure 5:**
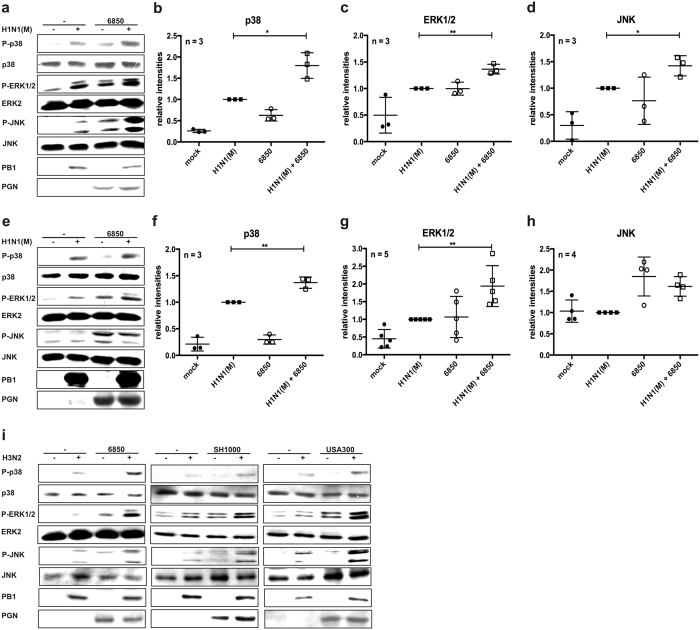
Phosphorylation of the MAPKs p38, ERK1/2 and JNK is increased upon IV/*S. aureus* super-infection. Calu-3 (**a**–**d**) or A549 (**e**–**i**) cells were infected with IV H1N1(M) (**a**–**h**) or H3N2 (**i**) (MOI 5) for 0.5 h and super-infected with *S. aureus* 6850 (**a**–**i**), SH1000 or MRSA USA300 (**i**) (MOI 50). Extracellular bacteria were removed by gentamicin treatment 3 h after bacterial infection. At 8 h p.i. cells were lysed and whole cell lysates were subjected to Western blot analysis monitoring P-p38, P-ERK1/2, P-JNK, PGN and PB1. Equal protein loading was verified by detection of p38, ERK2 and JNK (**a**,**e**,**i**). Original blots are shown in Supplementary Fig. S8b (upper panel: Fig. 5a, lower panel: Fig. 5e) and S8c (upper panel: Fig. 5i [S. *aureus* 6850], lower panel [*S. aureus* SH1000, USA300]). Relative levels of P-p38, P-ERK1/2 and P-JNK compared to p38, ERK2 or JNK bands, respectively, were quantified by using ImageJ 2006.02.01 software (**b**–**d**,**f**–**h**). Means ± SD of at least three independent experiments are shown. Two-tailed unpaired t-tests were performed for comparison of IV H1N1(M)-infected and IV H1N1(M)/*S. aureus* 6850 super-infected samples (*p < 0.05, **p < 0.01).

Various signalling pathways regulate the production of IL-6 in respiratory epithelial cells. Since secondary bacterial infection after IV infection induces hyper-phosphorylation and activation of several members of the MAPK family, namely JNK, p38 and ERK1/2^11^, the phosphorylation status of these MAPKs was investigated in more detail by Western blot analysis (Fig. 5).

Viral or bacterial infection with a single pathogen resulted in a moderate phosphorylation of the MAPKs p38, ERK1/2 and JNK in Calu-3 cells. Upon IV/*S. aureus* super-infection the most prominent effect was observed for the MAPK p38, where super-infection resulted in a strong synergistically enhanced phosphorylation of the kinase. However, phosphorylation levels of ERK1/2 and JNK were also found to be elevated in the presence of both pathogens (Fig. 5a–d). In A549 cells similar results were obtained for the MAPKs p38 and ERK1/2 (Fig. 5e–g). While MAPK JNK was not elevated in IV H1N1(M)-infected A549 cells (Fig. 5e,h), super-infection with other IV strains clearly resulted in higher JNK activity (Fig. 5i). This might be due to amino acid substitutions in the viral protein NS1 that influence MAPK JNK activity after IV infection in A549 cells^43^.

Based on these results we hypothesised that the synergistic hyper-activation of MAPK p38 and to a lesser extent of MAPKs ERK1/2 and JNK might be the reason for the hyper-induction of IL-6 after super-infection.

### Inhibition of MAPKs p38 and ERK1/2 reduces IV/*S. aureus*-induced *IL*-*6* mRNA levels

**Figure 6:**
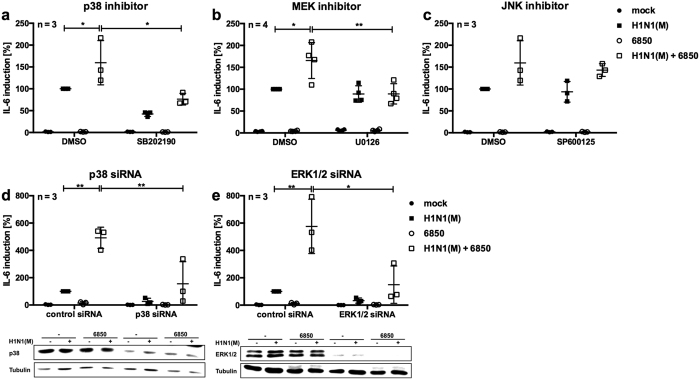
Specific inhibition of the MAPK p38 or the Raf/MEK/ERK signalling pathway reduces *IL*-*6* mRNA levels during super-infection. A549 cells were treated with 10 μM p38 inhibitor (SB202190) (**a**), 10 μM MEK inhibitor (U0126) (**b**), 10 μM JNK inhibitor (SP600125) (**c**) or DMSO as solvent control for 0.5 h. A549 cells were transfected with 33 nM siRNA directed against p38 (**d**), ERK1/2 (**e**) or control siRNA and incubated for 48 h. Subsequently, cells were infected with IV H1N1(M) (MOI 5) for 0.5 h and super-infected with *S. aureus* 6850 (MOI 50) (**a**–**e**), in the presence of the inhibitors (**a**–**c**). Extracellular bacteria were removed by gentamicin treatment 3 h after bacterial infection (**a**–**e**), and supplemented with the specific inhibitors (**a**–**c**). Expression of p38 (**d**) and ERK1/2 (**e**) was monitored by Western blot analysis (original blots are depicted in Supplementary Fig. S8d). *IL*-*6* mRNA levels were measured in duplicates at 8 h p.i. Means ± SD of at least three independent experiments are shown. IV-infected samples of control cells were arbitrarily set as 100%. After normalisation, one-way ANOVA followed by Dunnett’s multiple comparison tests were performed for comparison of IV H1N1(M)-infected and IV H1N1(M)/*S. aureus* 6850 super-infected samples, control and treated IV-infected samples, and control and treated super-infected samples (*p < 0.05, **p < 0.01).

To investigate a potential correlation between the hyper-induction of *IL*-*6* and increased activation of MAPKs p38, ERK1/2 and JNK during IV/*S. aureus* super-infection, specific inhibitors of these MAPKs were used. A549 cells were treated with the MAPK p38 inhibitor SB202190, the MEK inhibitor U0126 or the MAPK JNK inhibitor SP600125. *IL*-*6* mRNA levels were then measured by RT-qPCR after super-infection with IV H1N1(M) and *S. aureus* 6850 and compared with *IL*-*6* mRNA levels after treatment with solvent control (Fig. 6a–c). Inhibition of the MAPK p38 as well as treatment with the MEK inhibitor U0126 resulted in decreased *IL*-*6* mRNA levels in cells super-infected with IV/*S. aureus* (Fig. 6a,b). Inhibition of MAPK JNK with SP600125 had no effect on *IL*-*6* mRNA levels after challenge with a single pathogen or with both pathogens (Fig. 6c). To rule out any interfering effect of the MAPK inhibitors on viral replication, progeny virus titres of IV H1N1(M) were monitored by standard plaque assays. No significant differences between solvent control and inhibitor-treated cells were detectable at 8 h p.i. (data not shown).

To verify the results of the inhibitor studies described above, specific siRNAs against MAPKs p38 and ERK1/2 were used in super-infection experiments. Efficient knockdown was confirmed by Western blot analysis. Knockdown of either p38 or ERK1/2 resulted in reduced *IL*-*6* mRNA levels upon super-infection with IV H1N1(M) and *S. aureus* 6850 compared with control cells (Fig. 6d,e).

Consequently, *IL*-*6* mRNA expression is dependent on the activation of MAPKs p38 and ERK1/2 after infection of A549 cells.

### Phosphatase activity is reduced upon IV infection in A549 cells

**Figure 7:**
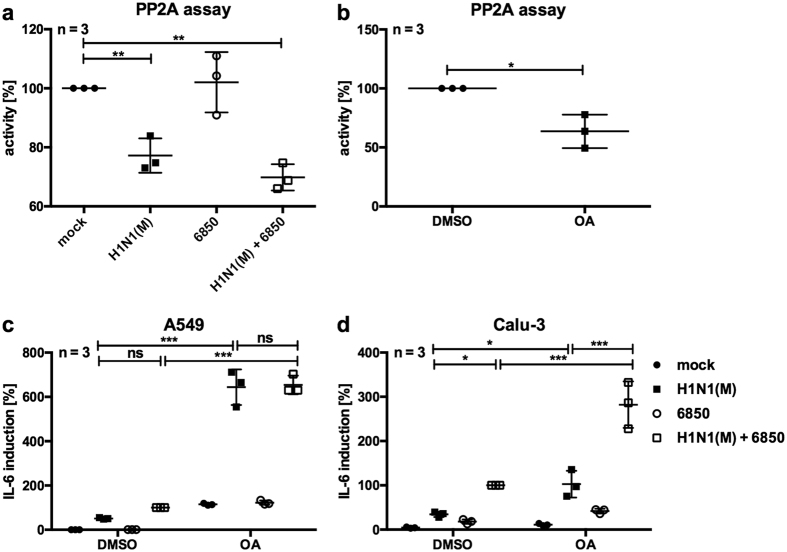
Phosphatase activity of PP2A is decreased upon IV H1N1(M) infection. A549 (**a**,**c**) or Calu-3 (**d**) cells were infected with IV H1N1(M) (MOI 5) for 0.5 h and super-infected with *S. aureus* 6850 (MOI 50) (**a**,**c**,**d**), in the presence of 1 μM (**b**,**c**) or 250 nM (**d**) OA and the solvent DMSO. Extracellular bacteria were removed by gentamicin treatment 3 h after bacterial infection (**a**,**c**,**d**), and then incubated with the inhibitor (**c**,**d**). A549 cells were incubated in the presence of 1 μM OA or solvent DMSO for 7.5 h (**b**). Cell lysates were subjected to PP2A activity assays according to the manufacturer’s instructions (**a**,**b**). *IL*-*6* mRNA levels were measured in duplicates at 8 h p.i. (**c**,**d**). Means ± SD of three independent experiments are shown. Mock-infected samples (**a**), DMSO treated (**b**) or DMSO treated super-infected (**c**,**d**) cells were arbitrarily set as 100%. After normalisation, one-way ANOVA followed by Dunnett’s multiple comparison tests were performed for comparison between mock-treated and IV H1N1(M)-infected or IV H1N1(M)/*S. aureus* 6850 super-infected samples, control- and OA-treated samples (*p < 0.05, **p < 0.01, ***p < 0.001).

Kinases that drive cellular inflammatory signalling events have to be tightly regulated and are usually counterbalanced by dephosphorylation mediated by a number of phosphatases. The protein phosphatase PP2A is a master controller of these signalling pathways, dephosphorylating MAPKs in A549 cells and thereby regulating IL-6 expression in a non-infection scenario^40^. To investigate a potential role of PP2A during IV/*S. aureus* super-infection, PP2A activity was measured using a phosphatase activity assay. Infection of A549 cells with IV H1N1(M) resulted in decreased PP2A activity, while bacterial infection did not affect the phosphatase activity. Super-infection with both pathogens decreased PP2A activity by 30% compared with mock-infected cells (Fig. 7a). Moreover, inhibition of PP2A activity by treatment with okadaic acid (OA) (Fig. 7b) resulted in increased *IL*-*6* mRNA levels of IV or IV/*S. aureus* super-infected A549 and Calu-3 cells compared with control cells (Fig. 7c,d).

These results indicate a connection between decreased PP2A activity and elevated IL-6 levels upon IV/*S. aureus* super-infection.

### IV/*S. aureus* super-infection increases IL-6 dependent APP levels *in vivo*

**Figure 8:**
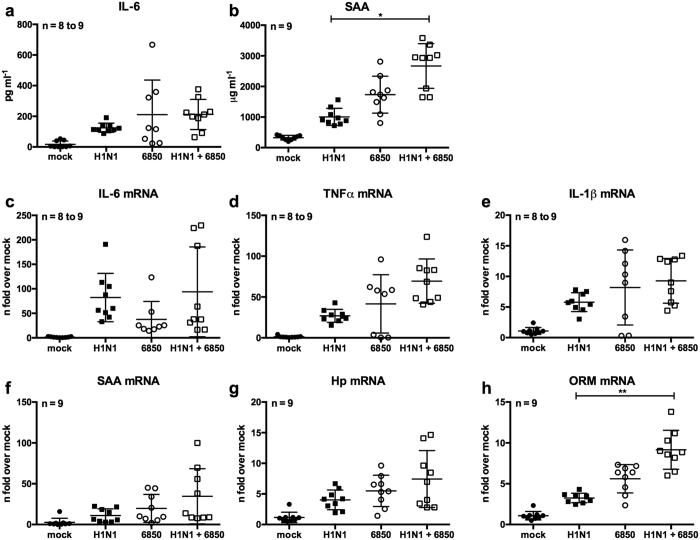
Infection with IV H1N1 and *S. aureus* 6850 induces an inflammatory response *in vivo*. Balb/c mice were intranasally infected with IV H1N1 (50 PFU) and were super-infected with *S. aureus* 6850 (5 × 10^7^ CFU) on day 2 post viral infection. 16 h post bacterial infection, animals were sacrificed, and blood, lung and liver samples were subjected to further investigation. Serum levels of IL-6 (**a**) and SAA (**b**) were analysed by ELISA. Total RNA of lung (**c**–**e**) and liver (**f**–**h**) samples was isolated and the levels of *IL*-*6* (**c**), *TNFα* (**d**), *IL*-*1β* (**e**), *SAA* (**f**), *Hp* (**g**) and *ORM* (**h**) were measured by RT-qPCR and are depicted as fold change over mock-treated animals. Mean ± SD is shown. Kruskal-Wallis followed by Dunnett’s multiple comparison tests were performed for comparison of single and super-infected animals (*p < 0.05, **p < 0.01).

In order to show the relevance of our *in vitro* findings in an *in vivo* super-infection model, Balb/c mice were infected with IV H1N1 and super-infected with *S. aureus* 6850. The amounts of IL-6 and APPs were measured at the mRNA and protein level. Infection with a single pathogen as well as super-infection with both pathogens increased IL-6 levels compared to mock-infected animals. However, only slight and not significant differences between groups challenged with a single pathogen or both pathogens were detected (Fig. 8a,c). Similar results were obtained for the pro-inflammatory cytokines TNFα and IL-1β (Fig. 8d,e). In contrast, the serum levels of the APP SAA and mRNA levels of *ORM*, whose expression is strongly dependent on IL-6 in mice and humans^28^,^29^, were significantly enhanced after super-infection compared to IV-infected mice (Fig. 8b,h). An alike tendency between IV and super-infected mice was detected in case of *SAA* and *Hp* mRNA levels (Fig. 8f,g). However, no significant differences were detected between *S. aureus* and super-infected animals (Fig. 8). Nevertheless, a similar increase in APPs was detected upon IV/*S. aureus* super-infection *in vitro* (Supplementary Fig. S1).

In summary, these results show a direct link between hyper-activity of the MAPKs p38 and ERK1/2 and an over-production of the multifunctional cytokine IL-6, which is promoted by decreased PP2A activity during IV/*S. aureus* super-infection. Additionally, increased IL-6 levels lead to higher expression of APPs after challenge with both pathogens.

## Discussion

*S. aureus* is one of the most common bacterial pathogens identified in patients with bacterial pneumonia during IV pandemics of the last one hundred years^44^,^45^. Recently, an increase in mortality associated with IV/*S. aureus* super-infections has been detected^5^,^7^. Hallmarks of severe infection are a dysregulation of immune responses accompanied by increased cytokine and chemokine expression, detrimental inflammation, and enhanced pathogen load^15^,^24^,^41^. In particular, elevated IL-6 levels correlated with disease severity in patients infected with the pandemic influenza strain in 2009, although super-infection with *S. aureus* was not confirmed in these cases^16^,^17^,^18^,^19^,^20^,^21^,^22^. Also, severe disease after seasonal IV infections is associated with increased IL-6 levels^23^. Thus, we focussed on the induction and regulation of the multifunctional cytokine IL-6 during IV/*S. aureus* super-infection.

In the present study we show that IV/*S. aureus* super-infection synergistically increases IL-6 levels at both the mRNA and protein levels in different cell lines at several time points of infection (Figs 1–3, Supplementary Fig. S2). This effect was not dependent on differences in pathogen load (Supplementary Fig. S3). A synergistic increase of IL-6 was independent of the IV and *S. aureus* strains used (Figs 2 and 4c), suggesting that expression of strain specific virulence factors is not responsible for IL-6 hyper-induction in epithelial cells. Under the same study conditions several pro-inflammatory cytokines and chemokines were increased upon *in vitro* infection. Surprisingly, IV-induced pro-inflammatory cytokines and chemokines were substantially elevated after super-infection with *S. aureus*, while *S. aureus*-induced *IL*-*1β* and *CXCL8* expression levels were not escalated by viral infection (Fig. 1). Additionally, expression of APPs was increased in hepatic cells upon challenge with conditioned medium of IV/*S. aureus* super-infected cells (Supplementary Fig. S1).

In infected cells, binding of PAMPs to innate immunity receptors triggers the inflammatory response. We showed that co-stimulation with the TLR2 ligand LTA induced hyper-transcription of *IL*-*6* mRNA in IV-infected cells, while other bacterial components and toxins failed to induce this synergistic effect (Fig. 4). Surprisingly, co-stimulation with HKSA, PKSA or EKSA had no effect on IV-induced *IL*-*6* expression (Fig. 4b,c), although LTA was still present. This might be due to LTA degradation during the inactivation process or interference with the LTA-induced signal by another bacterial component. A recent study demonstrated that binding of PGN-embedded molecules to TLR2 induces an anti-inflammatory instead of a pro-inflammatory response in T cells^46^. The dependence of IL-6 expression on TLR2 signalling during IV/*S. aureus* super-infection was further emphasised by knockdown of the adaptor protein MyD88 (Fig. 4f) or treatment with neutralising antibodies directed against TLR2 (Fig. 4h). Interestingly, a lethal inflammatory response against IV and secondary bacterial infection was previously linked to TLR2 signalling^13^. Furthermore, TLRs are marginally expressed on respiratory epithelial cells under physiological conditions, but TLR2 expression is increased upon infection^47^, which might worsen the course of disease.

It is well established that pathogen replication and host cytokine responses are controlled by pathogen-regulated signalling events. Several members of the MAPK family are activated upon IV or *S. aureus* infection and mediate distinct responses. The activity of MAPKs has been linked to the expression of inflammatory cytokines and chemokines such as IL-6, CCL5 and CXCL8^32^,^48^,^49^. Also, the dysregulation of the MAPK p38 induces hypercytokinaemia upon single infection with HPAIV^50^. Furthermore, the MAPK ERK1/2 signalling pathway is activated upon IV infection to promote replication by inducing nuclear export of viral RNPs^37^. Moreover, pro- and anti-inflammatory responses are induced after *S. aureus* infection via MAPK p38 and ERK1/2^38^. Here, we show for the first time that IV/*S. aureus* super-infection resulted in a synergistically enhanced phosphorylation of the MAPK p38 as well as increased activation of the MAPKs ERK1/2 and JNK in an *in vitro* cell culture model system (Fig. 5). Our data correlate with results that were previously described in an *in vivo* super-infection model using IV and *S. pneumoniae*^11^. Furthermore, by the use of several inhibitors and siRNAs, IL-6 expression upon IV/*S. aureus* super-infection was specifically linked to the activity of MAPKs p38 and ERK1/2 (Fig. 6).

The activity of MAPKs has to be strictly regulated under physiological conditions. Several classes of protein phosphatases have been shown to dephosphorylate and thereby negatively regulate MAPK activity, including tyrosine, serine/threonine and dual-specific MAPK phosphatases^51^. Here we detected that the activity of PP2A is decreased upon IV infection as well as upon IV/*S. aureus* super-infection (Fig. 7a), which might result in prolonged MAPK phosphorylation and increased cytokine expression. Inhibition of PP2A by OA treatment confirmed the link between PP2A activity and IL-6 expression (Fig. 7b–d). Additionally, the important function of PP2A in controlling IL-6 expression in A549 cells has been described previously^40^. However, the exact regulatory mechanism of IV-mediated PP2A activity in the presence or absence of *S. aureus* is still unknown.

A synergistic activation of the inflammatory response upon viral and bacterial super-infection was shown in several *in vivo* model systems at the level of inflammatory cytokines and chemokines^11^,^12^. In this study, we detected an increase in inflammatory mediators in an *in vitro* super-infection model. Furthermore, in an *in vivo* infection model we showed a similar tendency of increased pro-inflammatory cytokines upon IV/*S. aureus* super-infection (Fig. 8a,c–e). Previous studies revealed significantly different IL-6 levels between single and super-infected mice^52^,^53^. Interestingly, Robinson *et al*. reported that at an early stage a single *S. aureus* infection induced a stronger IL-6 expression than IV/*S. aureus* super-infection, but after 24 h of infection IL-6 levels in super-infected mice were higher than in single infected animals^53^. Nevertheless, we recognised significantly elevated APPs upon IV/*S. aureus* super-infection in mice (Fig. 8) as well as *in vitro* (Supplementary Fig. S1) indicating a strong induction of the pro-inflammatory response at very early stages of super-infection. It is a common observation that the levels of inducing cytokines are only marginally affected while the induced downstream effectors are significantly enhanced, e.g. this has been shown for type I IFN induction and expression of IFN-stimulated genes^54^. The APR is rapidly induced upon infection to limit pathogen spread and minimise tissue damage^26^. Intriguingly, a similar increase of APPs has been shown for infections with HPAIV^55^ and APPs can amplify the inflammatory response. For instance, SAA is a chemoattractant for inflammatory cells and stimulates the expression of pro-inflammatory cytokines and tissue degrading proteases^56^,^57^,^58^. These biological activities might enhance inflammation during IV/*S. aureus* super-infection and increase morbidity. So far, the outcome of elevated APPs during a super-infection scenario has not been explored in detail, but increased levels of SAA have already been linked to severe disease in an IV and *Pasteurella multocida* super-infection model^59^.

Taken together, these results reveal the molecular mechanism of dysregulated IL-6 expression upon IV/*S. aureus* super-infection in an *in vitro* model.

## Methods

### Cell lines, viral and bacterial strains

Madin-Darby canine kidney cells (MDCK) and the human bronchial epithelial cell line (Calu-3) were cultivated in minimal essential medium (MEM), the human alveolar basal epithelial cell line (A549) was cultivated in Dulbecco’s modified eagle medium (DMEM). Both media were supplemented with 10% foetal bovine serum (FBS).

The influenza virus strains A/Puerto Rico/8/34 (H1N1(M)) and B/Lee/40 (FluB) were propagated in 11-day-old embryonated chicken eggs, the influenza virus strains A/Wisconsin/67/2005 (H3N2), A/Hamburg/04/2009 (H1N1v), the recombinant A/Seal/Massachusetts/1/80 (H7N7) and the recombinant A/Puerto Rico/8/34 (H1N1) were passaged on MDCK cells.

The *S. aureus* strains 6850 (ATCC 53657), SH1000 and MRSA USA300 were cultivated as described previously^60^. To obtain heat- (HKSA), paraformaldehyde- (PKSA) or ethanol-killed (EKSA) bacteria, *S. aureus* 6850 was treated as described earlier^15^. Efficient inactivation was verified by plating on agar plates and incubating overnight at 37 °C.

### Mouse experiments

All animal studies were performed in compliance with the animal welfare regulations of the German Society for Laboratory Animal Science (GV-SOLAS) and the European Health Law of the Federation of Laboratory Animal Science Associations (FELASA). The protocol was approved by the State Agency for Nature, Environment and Consumer Protection (LANUV), Germany (permission number Az 84.02.04.2013 A289). Female Balb/c mice (Harlan Laboratories, Rossdorf, Germany), nine to eleven weeks old, were anaesthetised by isoflurane (Abbvie, Wiesbaden, Germany) inhalation and intranasally infected with IV (50 PFU) or PBS in a 50 μl volume. The same method was used for *S. aureus* (5 × 10^7^ CFU) infection two days later. The health status of the animals was monitored twice daily. 16 h after bacterial infection, animals were sacrificed and samples of blood, liver and lung were subjected to further analysis.

### Isolation of cellular and bacterial DNA

Bacterial DNA was isolated from 1 ml of a fresh over-night culture of *S. aureus* 6850, cellular DNA was isolated from 2 × 10^7^ A549 cells. Cells were pelleted for 5 min at 3200× g and 4 °C. The pellet was resuspended in 493 μl TE buffer [10 mM Tris/HCl, 1 mM EDTA, pH 8.0]. For lysis, cells were incubated with 4 μg ml^−1^ lysostaphin (Sigma, Munich, Germany) for 10 min at 37 °C. Subsequently, 2.4 μg ml^−1^ Proteinase K (Sigma) was added and the suspension was incubated for further 10 min at 37 °C. Lysis was stopped by incubation for 5 min at 95 °C. One volume of phenol/chloroform/isoamyl alcohol (Roth, Karlsruhe, Germany) was added and samples were centrifuged for 10 min at 20800× g. The upper aqueous phase was used to repeat the phenol/chloroform/isoamyl alcohol extraction step. The aqueous phase was transferred to a clean tube and mixed with 1/10 volume of 5 M NaCl and 6/10 volume of isopropanol. DNA was precipitated for 0.5 h at 20800× g, 4 °C. The pellet was washed once with 70% ethanol and centrifuged for 15 min at 20800× g and 4 °C. Finally, the pellet was air dried and dissolved in water. Bacterial DNA concentration and purity were determined by NanoDrop (PEQLAB Biotechnology, Erlangen, Germany) measurement.

### *In vitro* infection with IV and *S. aureus*

Cells were seeded in 6-well plates (A549: 0.75 × 10^6^, Calu-3: 1.5 × 10^6^) in 2 ml of culture medium or in 12-well plates (A549: 0.35 × 10^6^, Calu-3: 0.75 × 10^6^) in 1 ml culture medium 16 h (A549) or 40 h (Calu-3) prior to infection.

Cells were washed and incubated with 500 μl PBS/BA [0.2% bovine serum albumin (BSA), 1 mM MgCl_2_, 0.9 mM CaCl_2_, 100 U ml^−1^ penicillin, 0.1 mg ml^−1^ streptomycin] containing the virus with the indicated multiplicity of infection (MOI) at 37 °C, 5% CO_2_. After 0.5 h incubation, the suspension was aspirated, cells were washed with PBS and super-infected with the bacteria at the indicated MOI in 1 ml of DMEM/INV (A549) or MEM/INV (Calu-3) [the indicated media being supplemented with 1% human serum albumin, 25 nmol l^−1^ HEPES]. Subsequently, cells were incubated for 3 h at 37 °C, 5% CO_2_.

For incubation times >5 h, growth of extracellular bacteria was inhibited by an antibiotic wash. Thereafter, cells were washed with PBS and incubated with 1 ml of DMEM (A549) or MEM (Calu-3) supplemented with 10% FBS, 100 μg ml^−1^ gentamicin (Applichem, Darmstadt, Germany) or 2 μg ml^−1^ lysostaphin (Sigma) for 0.5 or 0.33 h, respectively, at 37 °C, 5% CO_2_. Treatment with lysostaphin was only performed in case of an overnight incubation. Subsequently, cells were washed and incubated in 1 ml of DMEM/BA (A549) or MEM/BA (Calu-3) [0.2% BSA, 1 mM MgCl_2_, 0.9 mM CaCl_2_] until the end of incubation time at 37 °C, 5% CO_2_.

### Cellular stimulation with IV and bacterial components

A549 cells were infected with IV as described above, washed with PBS and infected with *S. aureus* 6850, HKSA, PKSA or EKSA (MOI 50) or were incubated with 1 ml of DMEM/INV supplemented with 5 μg ml^−1^ LTA, 5 μg ml^−1^ PGN, 0.5 μg ml^−1^ SEA, 0.5 μg ml^−1^ SEB or DMSO for 7.5 h at 37 °C, 5% CO_2_. LTA, SEA and SEB were purchased from Sigma, PGN was purchased from InvivoGen (San Diego, USA). LTA and PGN were dissolved in water, SEA and SEB were dissolved in dimethylsulphoxide (DMSO).

For stimulation with cellular and bacterial DNA, cells were transfected, directly after IV infection, with 10 μg DNA using Lipofectamine®2000 (Invitrogen, Carlsbad, USA) according to the manufacturer’s protocol and incubated in DMEM/INV for 7.5 h at 37 °C, 5% CO_2_.

### Treatment with inhibitors or neutralising antibodies during infection with IV and *S. aureus*

U0126 was purchased from Taros GmbH (Dortmund, Germany) and okadaic acid (OA) was obtained from LC Laboratories (Woburn, USA). SB202190 and SP600125 were purchased from Sigma. All inhibitors were dissolved in DMSO. Cells were washed with PBS and incubated with 1 ml of culture medium supplemented with 10 μM SB202190, 10 μM U0126, 10 μM SP600125 or DMSO for 0.5 h at 37 °C, 5% CO_2_ prior to infection. Treatment with OA started after IV infection. Cells were infected with IV and *S. aureus* as described above. During incubation with DMEM/INV and DMEM/BA, media were additionally supplemented with the inhibitors at the indicated concentrations.

Neutralising antibodies for TLR2 and an isogenic control were purchased from InvivoGen and dissolved in water. Cells were infected with IV as described above and incubated for 3 h. Afterwards DMEM/INV containing 5 μg ml^−1^ antibodies was added for 0.5 h. Then, *S. aureus* was directly added into the medium and cells were further incubated for 4 h.

### Transfection of siRNA

Specific siRNA directed against MyD88 was purchased from Qiagen (Hilden, Germany). siRNAs against the MAPKs p38 and ERK1/2 were purchased from Cell Signalling Technology (Danvers, USA). All siRNAs were dissolved in water. 30 nM (MyD88) or 33 nM (MAPKs) and the corresponding control siRNA were transfected into A549 cells (12-well: 0.2 × 10^6^) using Lipofectamine®2000 (Invitrogen) according to the manufacturer’s protocol and incubated in culture medium for 48 h at 37 °C, 5% CO_2_.

### Quantitative real-time PCR

For quantitative real-time PCR (RT-qPCR), total RNA of infected cells, lung and liver homogenates was isolated as described previously^50^. Equal amounts of RNA were transcribed into cDNA using Revert AID H Minus Reverse Transcriptase (MBI Fermentas, St. Leon-Rot, Germany) and oligo-(dT)-primers (Eurofins MWG Operon, Ebersberg, Germany) according to the manufacturer’s protocol. Samples were analysed by RT-qPCR on a Stratagene Cycler (Agilent Technologies, Santa Clara, USA) using gene-specific primers (Supplementary Table S6) and Brilliant SYBR Green Mastermix (Agilent, Waldbronn, Germany) according to the manufacturer’s instructions. To show reproducibility between experiments and obvious differences between mock-, single- and super-infected samples of *IL*-*6* mRNA levels, raw Ct values of IL-6 in different cell lines are shown in Supplementary Table S7. Additionally, PCR efficiency was calculated from cDNA of Calu-3 cells^61^. Relative expression levels were compared with the reference gene Glyceraldehyde 3-phosphate dehydrogenase (GAPDH) and calculated according to the 2^−ΔΔCt^ method^62^. The IV-infected samples were arbitrarily set as 100% and values of other samples were normalised to that value.

### Western blot analysis

For Western blot analyses, cells were infected as described above. Subsequently, cells were lysed overnight at 4 °C with 1× sodium dodecylsulphate sample buffer^63^ and denatured for 10 min at 95 °C. Cellular proteins were monitored by usage of P-p38 (T180/Y182, 1:500), P-JNK (T183/Y185, 1:500) (BD Transduction Technology, San Jose, USA), P-ERK1/2 (T202/Y204, 1:1000), JNK (1:500), ERK1/2 (1:1000), MyD88 (1:1000) (Cell Signalling Technology), p38 (C-20, 1:500), ERK2 (C-14, 1:1000) (Santa Cruz Biotechnology, Dallas, USA) and α-Tubulin (DM1A, 1:1000) (Sigma) antibodies. Viral protein expression was detected with goat anti-PB1 antibody (vK-20, 1:200) (Santa Cruz Biotechnology) and bacterial PGN was monitored by mouse anti-PGN (1:500) antibody (BioRAD, Hercules, USA). Protein bands were visualised using a standard enhanced chemiluminescence reaction. The original blots are presented in Supplementary Figure S8. Relative levels of target proteins were quantified by using ImageJ 2006.02.01 software and normalised to the loading of control samples.

### Enzyme-linked immunosorbent assay (ELISA)

For detection of human IL-6, cells were infected with IV and *S. aureus* as described above. Supernatants were collected at 8 h p.i. and incubated in a 96-well microplate coated with anti-human IL-6 (purchased from RayBiotech, Norcross, USA) overnight at 4 °C with gentle shaking. For measurement of murine IL-6 and SAA, blood samples were allowed to clot for 2 h at room temperature and centrifuged for 20 min at 2000× g. Diluted serum samples were incubated in 96-well microplates (IL-6: R&D Systems, Minneapolis, USA, SAA: Immunology Consultants Laboratory, Portland, USA) according to the manufacturer’s protocol. The intensity of colour development was measured on a Spectromax M2 instrument (Molecular Devices, Sunnyvale, USA). Each sample was measured in duplicate and the protein amount was calculated by comparison to a standard curve.

### PP2A activity assay

A549 cells were seeded in 15 cm (1 × 10^7^) or 10 cm (2.5 × 10^6^) plates and infected as described above, or treated with 1 μM OA in culture medium. 8 h p.i. or 7.5 h after treatment, cells were lysed and PP2A activity was measured by use of the PP2A immunoprecipitation phosphatase assay kit (Merck Millipore, Darmstadt, Germany), according to the manufacture’s instructions.

### Statistical analysis

Data are presented as means ± standard deviation (SD). Testing for normality was performed with the Shapiro-Wilk test. Statistical significance was evaluated using two-tailed unpaired t-tests, one-way analysis of variance (ANOVA) or Kruskal-Wallis analysis followed by Dunnett’s multiple comparison tests as indicated. A p value < 0.05 indicated a statistically significant difference.

## Additional Information

**How to cite this article:** Klemm, C. *et al*. Mitogen-activated protein kinases (MAPKs) regulate IL-6 over-production during concomitant influenza virus and *Staphylococcus aureus* infection. *Sci. Rep.* **7**, 42473; doi: 10.1038/srep42473 (2017).

**Publisher's note:** Springer Nature remains neutral with regard to jurisdictional claims in published maps and institutional affiliations.

## Supplementary Material

Supplementary Information
