# Supplementary material for: Mitogen-activated protein kinases (MAPKs) regulate IL-6 over-production during concomitant influenza virus and Staphylococcus aureus infection
Source: Sci Rep. 2017 Feb 14;7:42473. doi: 10.1038/srep42473 (PMC5307969; doi:10.1038/srep42473)
Supplement: Supplementary Information [file srep42473-s1.pdf]

Mitogen-activated protein kinases (MAPKs) regulate IL-6 over-production during concomitant influenza virus and *Staphylococcus aureus* infection

Carolin Klemm[1], Christin Bruchhagen[1], Andre van Krüchten[1], Silke Niemann[2], Bettina Löffler[3], Georg Peters[2,4], Stephan Ludwig[1,4], Christina Ehrhardt[1,4]*

[1] Institute of Virology Muenster (IVM), Westfaelische Wilhelms-University Muenster, Von Esmarch-Str. 56, D-48149 Muenster, Germany

[2] Institute of Medical Microbiology, University Hospital of Muenster, Domagkstr. 10, D-48149 Muenster, Germany

[3] Institute of Medical Microbiology, University Hospital Jena, Erlanger Allee 101, D-07747 Jena, Germany

[4] Cluster of Excellence Cells in Motion (CIM), University of Muenster, Muenster, Germany

* Corresponding author
PD Dr. rer. nat. Christina Ehrhardt
Phone: +49 (0)251-8353010
Fax: +49 (0)251-8357793
e-mail: ehrhardc@uni-muenster.de

Supplementary

Methods

Additional cell lines and bacterial strain

The human hepatic cell line HepG2 (a kind gift from Ute Albrecht, University of Duesseldorf, Germany) was cultivated in DMEM/F-12 medium 1:1. The human monocytic cell line THP-1 was cultivated in RPMI 1640 with 1% glutamine. The murine lewis lung carcinoma (LLC) and mouse embryonic fibroblast (MEF) cell lines were cultivated in DMEM. Each medium was supplemented with 10% FBS. Primary human umbilical vein endothelial cells (HUVEC) were cultivated in endothelial cell growth medium with supplement mix (PromoCell, Heidelberg, Germany).

THP-1 cells (6-well: 2×10^6, 12-well: 1×10^6) were stimulated with 10 ng ml^{-1} phorbol-12-myristate-13-acetate (PMA, AppliChem, Darmstadt, Germany) for differentiation into macrophages 16 h prior to infection. LLC (6-well: 0.3×10^6) and MEF cells (6-well: 0.25×10^6) were seeded 16 h and HUVECs (6-well: 0.5×10^6) were seeded 48 h prior to infection. During infection culture medium supplemented as indicated in the method section was used.

The mouse-adapted *S. aureus* strain LS1 was cultivated as described in the method section.

Plaque assay

Standard plaque titration was performed as described earlier[50].

Transfer of conditioned medium

HepG2 cells (12-well: 0.3×10^6) were seeded in 1 ml culture medium 40 h prior to stimulation. Calu-3 cells were infected as described in the method section. Supernatants of three 6-wells were pooled and sterile filtrated with a 0.2 µm filter to avoid transfer of living pathogens. HepG2 cells were washed and stimulated with 800 µl conditioned medium for 20 h at 37°C, 5% CO_2.

Actinomycin D treatment

A549 cells were infected with IV H1N1(M) (MOI 5) and *S. aureus* 6850 (MOI 50) as described in the material and method section. At 6 h p.i. cells were lysed, or washed and incubated in 1 ml of DMEM/BA supplemented with 1 µg ml^{-1} actinomycin D (Santa Cruz Biotechnology, Dallas, USA) for further 0.5, 1 or 2 h at 37°C, 5% CO_2. Cells were lysed and total cellular RNA was isolated as described in the method section. For investigation of *IL-6* mRNA stability, the amount of *IL-6* mRNA was measured for each time point indicated by RT-qPCR and *IL-6* levels were compared to untreated cells (0 h).

Stimulation of cells with recombinant human IFNβ

A549 cells were incubated in 1 ml of DMEM/INV with *S. aureus* 6850 (MOI 50) for 3 h at 37°C, 5% CO_2. Extracellular bacteria were removed by gentamicin treatment for 0.5 h at 37°C, 5% CO_2. Cells were incubated in 1 ml of DMEM/BA supplemented with 100 U recombinant human IFNβ (PBL Assay Science, Piscataway, USA) or same volume of PBS for 4.5 h at 37°C, 5% CO_2.

Supplementary figures

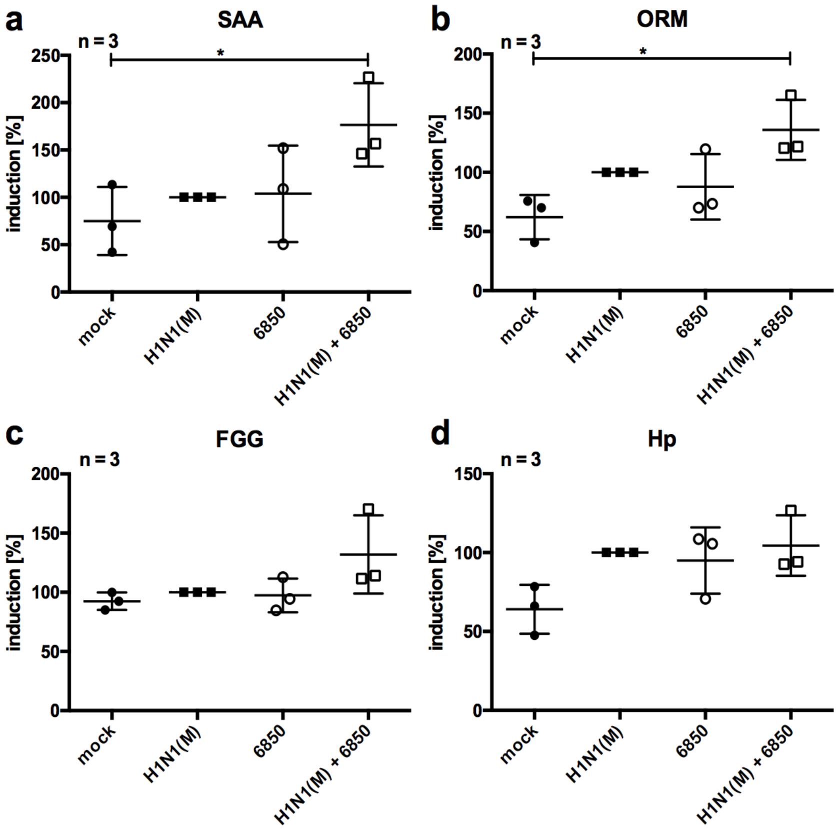

Supplementary figure S1: Conditioned medium of IV and *S. aureus* super-infected Calu-3 cells induces expression of APPs in HepG2 cells. Calu-3 cells were infected with IV H1N1(M) (MOI 5) for 0.5 h and super-infected with *S. aureus* 6850 (MOI 50). Extracellular bacteria were removed by gentamicin treatment 3 h post bacterial infection. At 8 h p.i. cell supernatants were sterile filtrated and added to HepG2 cells for another incubation of 20 h. Levels of *SAA* (a), *ORM* (b), *FGG* (c) and *Hp* (d) were measured in duplicates. Means ± SD of three independent experiments are shown. Samples, which were treated with conditioned medium of IV-infected Calu-3 cells, were arbitrarily set 100%. After normalisation, one-way ANOVA followed by Dunnett's multiple comparison tests were used to compare mock and IV and/or *S. aureus* infected samples (* $p < 0.05$).

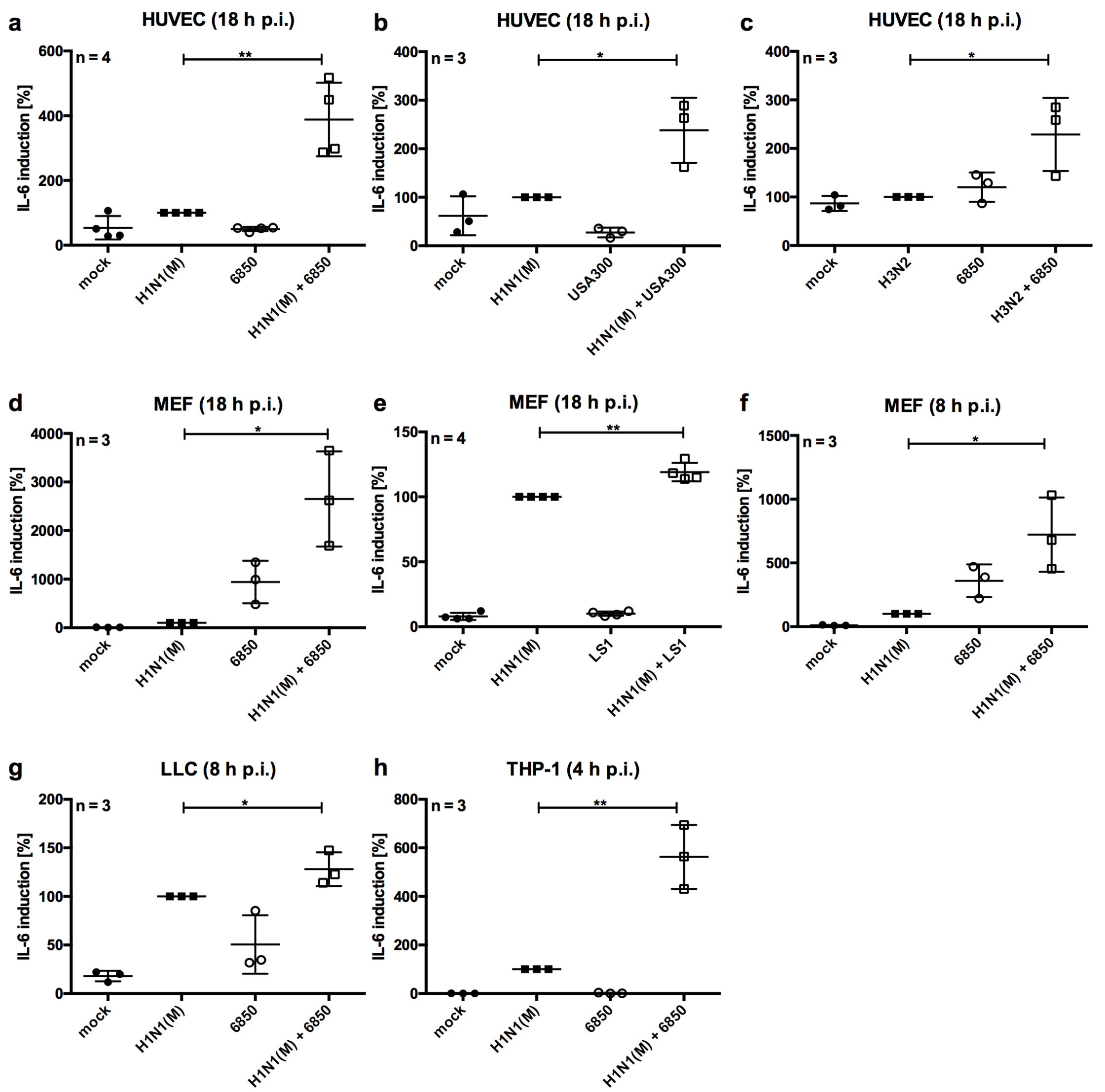

Supplementary figure S2: Increased *IL-6* expression during super-infection is cell line independent.

HUVEC (a – c), MEF (d – f), LLC (g) or THP-1 (h) cells were infected with H1N1(M) (MOI 0.5 (a, b, d, e), MOI 3 (f – h)) or H3N2 (MOI 0.5 (c)) for 0.5 h and super-infected with *S. aureus* 6850 (MOI 0.1 (a, c, d), MOI 10 (f, g), MOI 1 (h)), MRSA USA300 (b) or LS1 (e) (MOI 0.01). Extracellular bacteria were removed by antibiotic treatment 3 h post bacterial infection. *IL-6* mRNA levels were measured at 18 h (a – e), 8 h (f, g) or 4 h p.i. (h). Means ± SD of at least three independent experiments are shown. Levels of IV-infected samples

were arbitrarily set as 100%. After normalisation, two-tailed unpaired t-tests were performed for comparison between IV-infected and IV/*S. aureus* super-infected samples (* $p < 0.05$, ** $p < 0.01$).

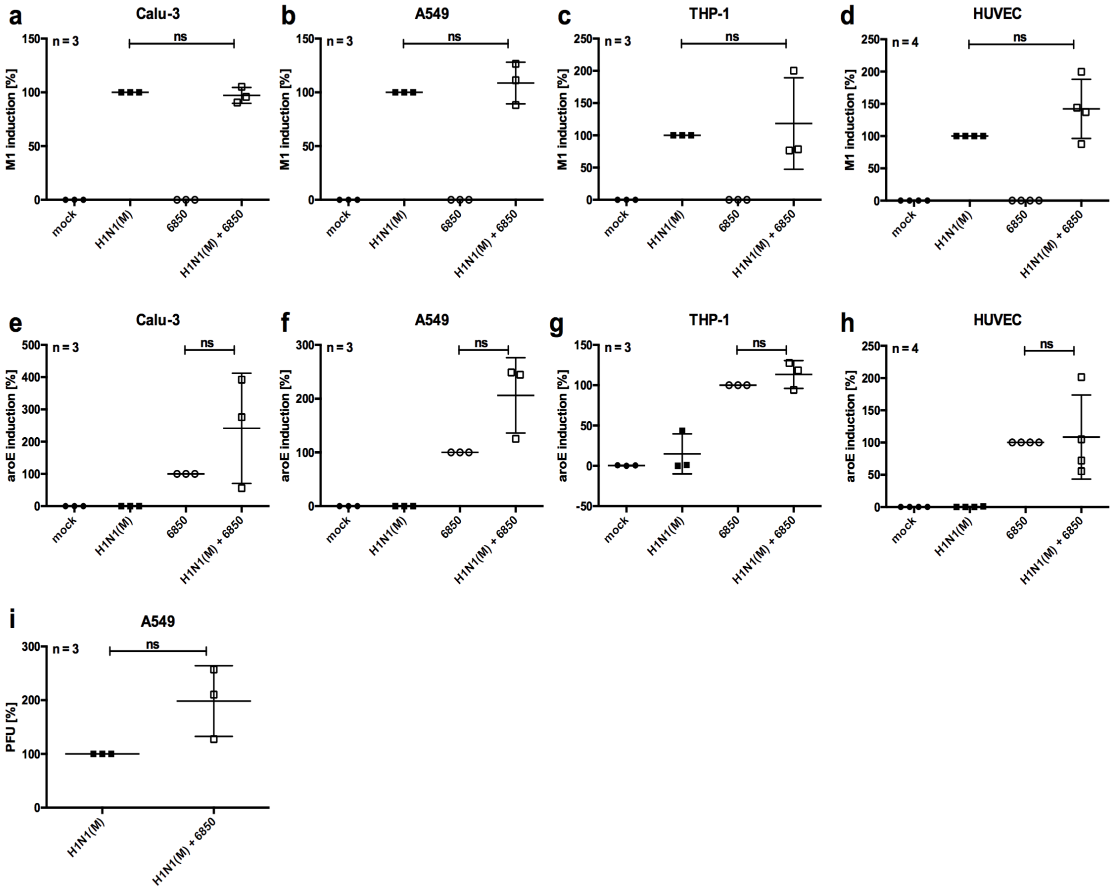

Supplementary figure S3: Levels of viral *M1* and bacterial *aroE* are not significantly changed upon super-infection with IV and *S. aureus*.

Calu-3 (a, e), A549 (b, f, i), THP-1 (c, g) or HUVEC (d, h) cells were infected with H1N1(M) (MOI 5 (a, b, e, f, i), MOI 3 (c, g), MOI 0.5 (d, h)) and super-infected with *S. aureus* 6850 (MOI 50 (a, b, e, f, i), MOI 1 (c, g), MOI 0.1 (d, h)). Extracellular bacteria were removed by antibiotic treatment 3 h post bacterial infection. *M1* (a – d) and *aroE* (e – h) levels were measured at 8 h (a, b, e, f), 4 h (c, g) or 18 h p.i. (d, h). Infectious virus titres were determined by standard plaque assay at 8 h p.i. (i). Means ± SD of at least three independent experiments are shown. Levels of IV (a – d, i) or *S. aureus* (e – h) infected samples were arbitrarily set as 100%. After normalisation, two-

tailed unpaired t-tests were performed for comparison between single and super-infected samples (ns not significant).

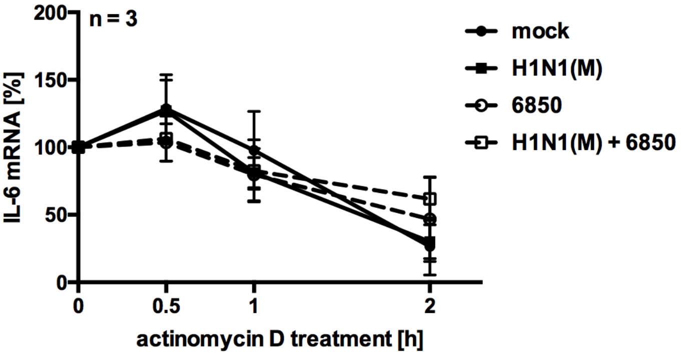

Supplementary figure S4: *IL-6* mRNA stability is not altered by infection or super-infection with IV H1N1(M) and/or *S. aureus* 6850.

A549 cells were infected with IV H1N1(M) (MOI 5) for 0.5 h and super-infected with *S. aureus* 6850 (MOI 50). Extracellular bacteria were removed by gentamicin treatment 3 h post bacterial infection. At 6 h p.i. cells were lysed or treated with 1 µg ml^{-1} actinomycin D for 0.5, 1 or 2 h. *IL-6* mRNA levels were measured in duplicates by RT-qPCR. Means ± SD of three independent experiments are shown. Untreated samples (0 h) were arbitrarily set as 100%.

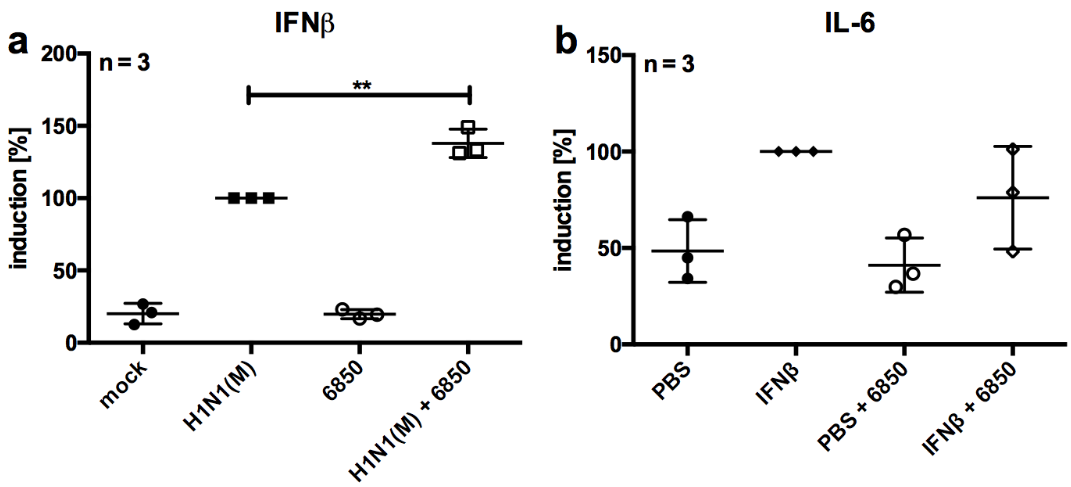

Supplementary figure S5: Increased IFNβ synthesis after super-infection does not induce hyper-transcription of *IL-6*.

A549 cells were infected with IV H1N1(M) (MOI 5) for 0.5 h and super-infected with *S. aureus* 6850 (MOI 50) (a) or cells were directly incubated with *S. aureus* 6850 (MOI 50) (b). Extracellular bacteria were removed by gentamicin treatment 3 h post bacterial infection. Subsequently cells were left untreated (a) or were stimulated with 100 U of recombinant human IFNβ or PBS as control (b). *IFNβ* (A) and *IL-6* (b) mRNA levels were measured in duplicates at 8 h p.i.. Means ± SD of three independent experiments are shown. IV-infected (a) or IFNβ-stimulated (b) samples were arbitrarily set as 100%. After normalisation, two-tailed unpaired t-test was performed for comparison of IV H1N1(M)-infected and IV H1N1(M)/*S. aureus* 6850 super-infected samples (** $p < 0.01$).

Supplemental table S6: Primer sequences for RT-qPCR

The tables list the sequences of used RT-qPCR primers.

human

Gene	Forward (5' – 3')	Reverse (5' – 3')
CCL3	AGTTCTCTGCATCACTTGCTG	CGGCTTCGCTTGGTTAGGAA
CCL5	CGGCACGCCTCGCTGTCATC	GCAAGCAGAAACAGGCAAAT
CXCL8	ACTGAGAGTGATTGAGAGAGTGGAC	AACCCTCTGCACCCAGTTTTC
FGG	GACGCTGCTACTTTGAAGTCC	TGGATTTGCACCGTGTCTTTG
GAPDH	GCCAATTCCATGGCACCGT	GCCCCACTTGATTTTGGAGG
Hp	CAGCACAGTCCCCGAAAAGAA	CAGTCGCATACCAGGTGTCC
IL-1β	CAGCTACGAATCTCCGACCAC	GGCAGGGAACCAGCATCTTC
IL-6	AACCTGAACCTTCCAAAGATGG	TCTGGCTTGTTCCTCACTAGT
IFNβ	TCTGGCACAACAGGTAGTAGGC	GAGAAGCACAACAGGAGAGCAA
SAA	CAAATACTTCCATGCTCGGGG	CGCAGCCTCTAACTTCTCCAC
ORM	GCTGTTCCTTAGGGACACCAA	TGACATCTGACCTGGGAATGC
TNFα	ATGAGCACTGAAAGCATGATC	GAGGGCTGATTAGAGAGAGGT

murine

Gene	Forward (5' – 3')	Reverse (5' – 3')
GAPDH	TGACCACAGTCCATGCCATC	GACGGACACATTGGGGGTAG
Hp	GCTATGTGGAGCACTTGGTTC	CACCCATTGCTTCTCGTCGTT
IL-1β	GCAACTGTTCCTGAACTCAACT	ATCTTTTGGGGTCCGTCAACT
IL-6	TAGTCCTTCCTACCCCAATTT	TTGGTCCTTAGCCACTCCTTC
ORM	CGAGTACAGGCAGGCAATTCA	ACCTATTGTTTGAGACTCCCGA
SAA	TGGCTGGAAAGATGGAGACAA	AAAGCTCTCTCTTGCATCACTG
TNFα	CCCTCACACTCAGATCATCTTCT	GCTACGACGTGGGCTACAG

pathogenic

Gene	Forward (5’ – 3’)	Reverse (5’ – 3’)
aroE	CTATCCACTTGCCATCTTTTAT	ATGGCTTTAATATCACAATTCC
M1	TGCAAAAACATCTTCAAGTCTCTG	AGATGAGTCTTCTAACCGAGGTCG

Supplemental table S7: Raw Ct values of *IL-6* in different cell lines upon infection

Analyses of *IL-6* mRNA were determined with Brilliant SYBR Green Mastermix (Agilent, Waldbronn, Germany) by RT-qPCR on a Stratagene Cycler (Agilent Technologies, Santa Clara, USA). Means and SD of at least three independent experiments with at least two biological replicates are shown. Ct values <35 were considered as specific.

		sample							
		**mock**		**H1N1(M)**		**6850**		**H1N1(M) + 6850**	
**cell line**	n	mean	SD	mean	SD	mean	SD	mean	SD
Calu-3 (8 h p.i.)	16	22.34	0.73	17.44	0.69	19.98	0.93	15.86	1.20
A549 (8 h p.i.)	24	30.38	0.89	23.71	0.53	28.62	1.30	22.00	0.49
THP-1 (4 h p.i.)	12	32.93	1.52	25.08	0.81	30.87	1.75	22.52	0.87
HUVEC (18 h p.i.)	16	22.34	1.40	21.68	1.86	22.26	1.55	20.04	0.91

A 2-fold dilution series of cDNA from Calu-3 cells was used for PCR efficiency (E) calculation[61]. Both primer pairs for IL-6 and GAPDH show high amplification efficiency, with an estimated E of 98.5% (± 1.8%) and 106.2% (± 6.1%), respectively.

a

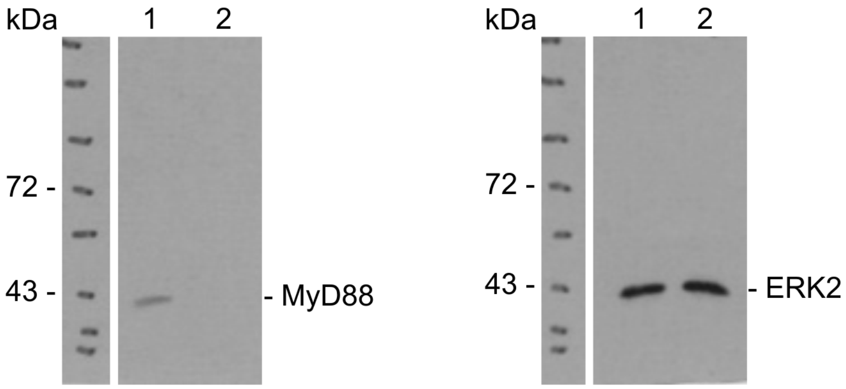

b

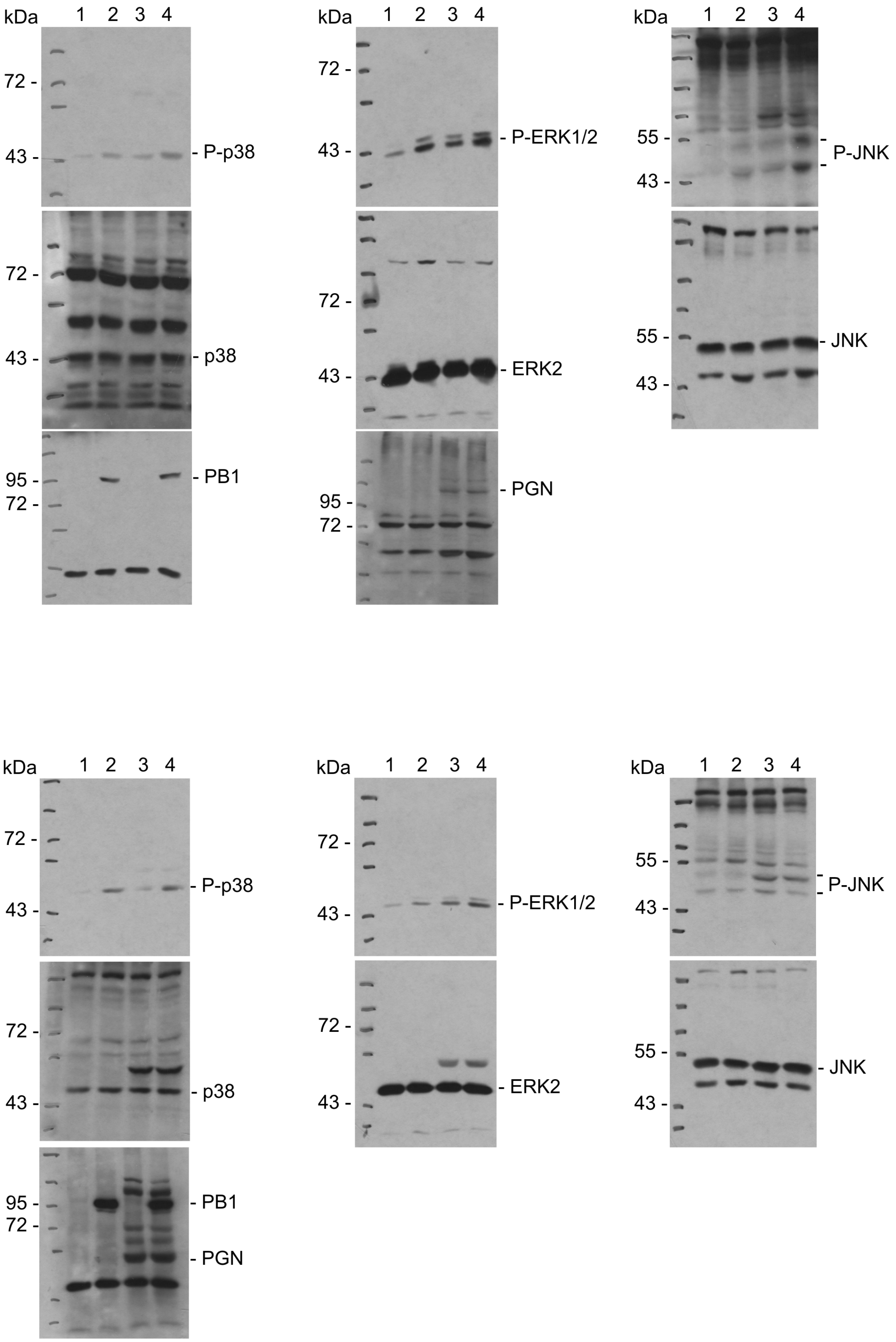

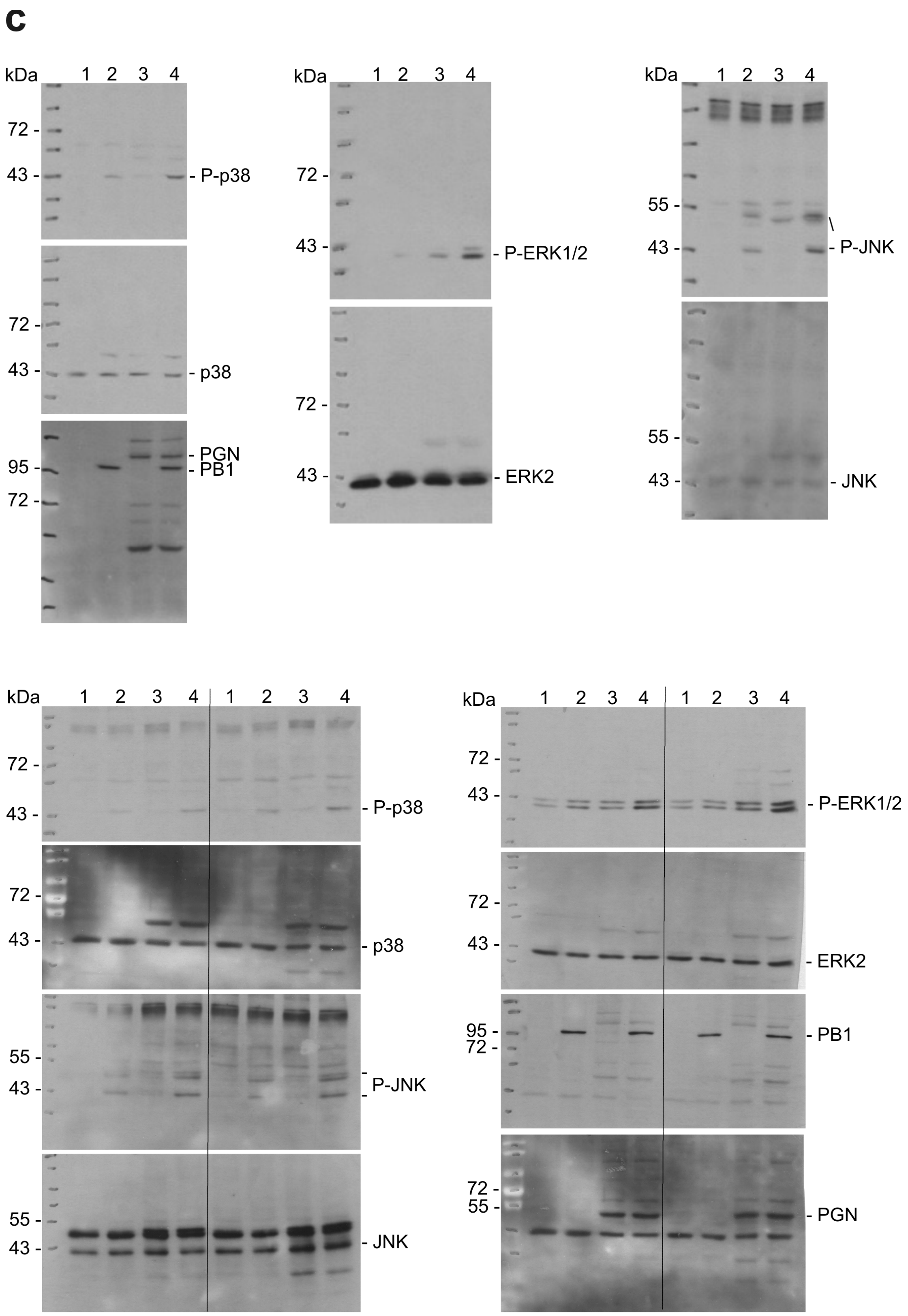
c
kDa
1
2
3
4
72 -
43 -
- P-p38
- p38
- PGN
- PB1
95 -
- P-ERK1/2
- ERK2
55 -
- P-JNK
- JNK

d

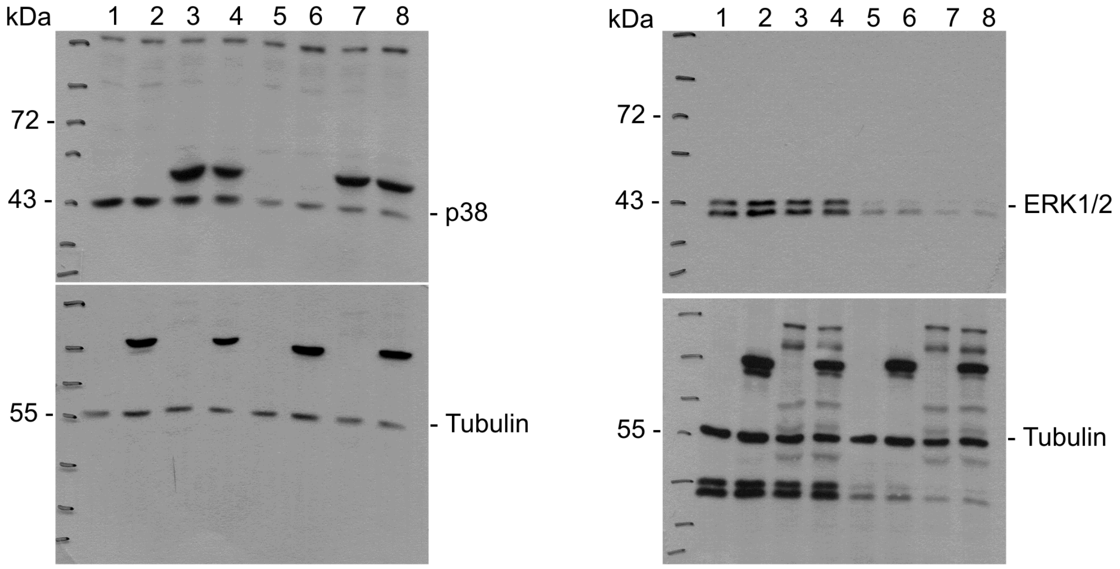

Supplementary figure S8: Original blots of figures 4g, 5a, e, i and 6d, e. Lane 1: mock-infected, lane 2: IV infection, lane 3: bacteria infection, lane 4: super-infection (a-d). In figure S8d lanes 5 - 8 represent the same infection scheme as described above, but in a siRNA scenario.

Figure S8a represents the original blots of figure 4g. Within figure S8b the upper panel shows the original blots of figure 5a, the lower panel represents the original blots of figure 5e. The upper panel of figure S8c corresponds to figure 5i (experiments performed with *S. aureus* 6850). On the left-hand side of the lower panel original blots of figure 5i (experiments performed with *S. aureus* SH1000) are shown. Blots on the right-hand side stand for figure 5i (experiments performed with *S. aureus* USA300).
